# NEAT1/miR-23a-3p/KLF3: a novel regulatory axis in melanoma cancer progression

**DOI:** 10.1186/s12935-019-0927-6

**Published:** 2019-08-22

**Authors:** Fei Ding, Jindong Lai, Yang Gao, Genhui Wang, Jingwen Shang, Daojun Zhang, Shumao Zheng

**Affiliations:** 1 Department of Dermatology, Zhoukou Central Hospital, Zhoukou, 466000 Henan China; 2 Department of Dermatology, Suining First People’s Hospital, Suining, 629000 Sichuan China; 3 Department of Dermatology, Affiliated Hospital of Hebei Academy of Traditional Chinese Medicine, Shijiazhuang, 050000 Hebei China; 4 Department of Dermatology, Hebei Provincial Hospital of Traditional Chinese Medicine, Shijiazhuang, 050000 Hebei China; 5 Department of Dermatology, The Third Affiliated Hospital of Chongqing Medical University, Chongqing, 400040 China; 6 Department of Dermatology, Hebei Academy of Chinese Medicine, Shijiazhuang, 050000 Hebei China

**Keywords:** NEAT1, miR-23a-3p, KLF3, Melanoma cancer

## Abstract

**Background:**

Melanoma is an extremely aggressive malignant skin tumor with high mortality. Many types of long noncoding RNAs and microRNAs have been reported to be associated with the oncogenesis of melanoma. However, a novel lncRNA-NEAT has not been thoroughly investigated in melanoma cancer. The purposes of this study were to investigate the underlying molecular mechanism in a novel couple of lnc-NEAT1 and miR-23a-3p, as well as the function role of KLF3 in the regulation of melanoma cancer.

**Methods:**

28 groups of tumor tissues and normal tissues were obtained from melanoma cancer patients. We performed a series of experiments and analysis, including RT-qPCR, western blots, CCK-8 assay, and migration/invasion assay, to investigate the expressions of NEAT1, miR-23a-5p and KLF3, cell viabilities, and tumor growth in vivo.

**Results:**

In this study, we observed that the expression of NEAT1 was significantly upregulated in melanoma tissues, which remarkedly promoted the cells’ proliferation, cell migration, and invasion in melanoma cell lines. Besides, NEAT1 could directly bind to miR-23a-3p, which was found to reverse the effect caused by NEAT1. MiR-23a-3p was discovered to bind to 3′UTR of KLF3, which reduced KLF3 expression. In addition, the overexpression of KLF3 could lower the effects of miR-23a-3p caused on melanoma cancer cell development.

**Conclusion:**

Our results demonstrated that NEAT1 could sponge miR-23a-3p and functions via the expression of KLF3. This axis of NEAT1/miR-23a-5p/KLF3 could together regulate melanoma cancer proliferation. This might provide a new therapeutic strategy for melanoma skin cancer.

*Trial registration* HBTCM38574839, registered 12 October 2012

## Background

Melanoma cancer is one of the most harmful skin cancers, usually originated from ultraviolet exposure from sunshine or tanning beds [1, 2]. In recent years, there are more than 150,000 persons have been diagnosed with melanoma cancer, with over 30% cases to be invasive and dangerous [3, 4]. Therefore, the early diagnostic and suitable treatment is critical and urgently required for melanoma skin cancer. In recent years, scientists have revealed the long-non-coding RNAs, lncRNAs, are playing important roles in tumorigenesis development or regulation [5–7]. They usually appear as an oncogene in many types of human cancers, including gastric cancer [8], lung cancer [9], breast cancer [10], and melanoma cancer [11]. Although many efforts have been paid, the numbers of lncRNAs for melanoma cancer are quite limited, with a huge space to explore.

NEAT1, nuclear enriched abundant transcript 1, is a novel lncRNA, transcribed from multiple endocrine neoplasia locus [8, 9, 12–17]. Previous studies have demonstrated that NEAT1 was continuously expressed in many cancer cell lines, such as lung cancer [9] and gastric cancer [8]. In 2018, Chen et approved that NEAT1 could be regulated by EGFR and contribute to glioblastoma progression [12]. There is a growing interest in the structural role of this novel lncRNA-NEAT1 in other types of cancers, for example, in melanoma skin cancer.

Recently, microRNAs are attracting great attention in its regulation of gene expression in tumor development [18, 19]. MiR-23a-3p is an important member of the microRNAs family. In 2019, F. Chen found that miR-23a-3p suppressed cell proliferation in oral squamous cell carcinomas (OSCC) and inhibited its growth in vivo [20]. For this specific microRNA of miR-23a-3p, it is essential to understand the detailed knowledge and their biological role of its functioning pathway, interaction with some lncRNAs, proteins, and other genes in the tumorigenesis.

Krüppel-like factor 3 (KLF3) is a protein expressed majorly in the red blood cell or erythroid lineage [6, 21–27]. Previous studies have found that the knockdown of this gene could result in the depression of some target genes. KLF3 was found to regulate lung cancer progression through the interaction with miR-326, and the regulatory axis of miR‐326/Sp1/KLF3 in 2018 [26]. Previous studies have established that KLF3 was a target gene of some specific miRNAs [26], while the miRNA could also be sponged by the corresponding lncRNAs [6, 28]. Through their targeting effect, they can form an axis of lncRNA/miRNA/mRNA to and exert the regulation effect in the progression, migration, and invasion of human cancer cells [6, 26, 28].

In this study, we aim to investigate the underlying molecular mechanism of NEAT1 and miR-23a-3p philologically, as well as the function role of KLF3 in melanoma cancer. We obtained 28 groups of tumor tissues and normal tissues, and performed a series of experiments and analysis, such as RT-PCR, western blots, CCK-8 assay, and migration/invasion assay, to examine the expressions, cell viabilities, and tumor growth in vivo. Our results found that in melanoma skin cancer (i) NEAT1 was over-expressed, and it promoted cell proliferation, migration, and invasion; (ii) expression of miR-23a-3p was inhibited by NEAT1; (iii) the sponge between NEAT1 and miR-23a-3p could regulate melanoma proliferation; and (iv) MiR-23a-3p targeted KLF3 and NEAT1/miR-23a axis regulated melanoma proliferation, migration, and invasion via KLF3. In summary, our study demonstrated that the oncogene NEAT1 could sponge miR-23a-3p, which would probably provide a new therapeutic strategy for melanoma skin cancer.

## Methods and materials

### Patients and clinical samples

In our study, we obtained 28 malignant melanoma patients. Before the surgery, they haven’t got any treatment of chemotherapy or radiotherapy. We identified the tumor samples through their histological diagnosis. We got the signed written informed consent from all the patients. Besides, we received the ethical approval for this study from the Hebei Provincial Hospital of Traditional Chinese Medicine.

### Cell lines and culture

We bought the human melanoma cell lines of M14, 451LU, A875, A375, and A2058 from the American Type Culture Collection (ATCC, USA). We cultured the cell lines in Dulbecco’s modified Eagle’s medium (DMEM, USA) accompanied with 10% fetal bovine serum (FBS, USA) and 100 U/ml penicillin/streptomycin. We purchased the human epidermal melanocytes neonatal cells (HEMn) from the Type Culture Collection of the Chinese Academy of Sciences (Shanghai, China). We cultured HEMn cells in melanocyte growth media (PromoCell, China). The culture was incubated at 37 °C with 5% CO_2_.

### Cell transfection

We synthesized multiple synthetic interfering RNAs (siRNA) and mimic oligonucleotide sequences targeting NEAT1 and miR-23a-3p from GenePharma Co. Ltd. (Shanghai, China). We performed the transfection through Lipofectamine 2000 transfection reagent, through strictly following the manufacturer’s instructions. GenePharma provided the oligonucleotide sequences.

### Quantitative real-time PCR (qRT-PCR)

We isolated total RNAs from (i) the melanoma tissues, (ii) their adjacent normal tissues, and (iii) Cell lines through TRIzol reagent (Invitrogen, USA). We synthesized cDNA from primers and their corresponding total RNA by RevertAid First Strand cDNA Synthesis Kit (Thermo Fisher, USA). Then, we performed qRT-PCR through an SYBR-Green PCR Master Mix Kit (Takara, China). We normalized the values for target gene expression to GAPDH and quantified the level of relative expression in the control group.

### Cell proliferation and colony formation assay

We evaluated cell viability through the cell counting kit-8 (CCK-8 assay) (Dojindo, Japan). Briefly, we plated 5 × 10^3^ cells in each well of the 96-well plates. We cultured the cells for 4 days. In each day, we measured their optical absorbance by a microplate reader (Bio-Rad, Hertfordshire, UK) at 450 nm. Then, we cultured melanoma cells (M14 and A375) in 6-well plates for 2 weeks. Finally, we fixed the cell colonies and stained them with 10% crystal violet. We counted the numbers using a microscope. More importantly, we conducted all the assays in triplicate.

### Dual-luciferase reporter assay

We amplified the 3′-UTR sequence of NEAT1 and KLF3 in normal human genomic DNA. Then, we subcloned the cells in pRL-CMV luciferase reporter vector (Ambion, USA). We seeded HEK293T cells at a density of 5 × 10^3^ cells in each well of the 96-well plates. Then, we co-transfected the cells with firefly luciferase target reporter and pRL-CMV Renilla luciferase control reporter. We co-transfected the miRNA mimics group or negative control group using Lipofectamine 2000 (Invitrogen, USA). We incubated the assay for 48 h and evaluated the luciferase activity through the Dual-Luciferase System (Promega, USA).

### Protein preparation and immunoblotting

We got the total protein extracts by a boiling buffer with 0.125 M Tris/HCl, and 2.5% sodium dodecyl sulphate at pH 6.8. We separated 30 μg proteins through sodium dodecyl sulphate polyacrylamide gel electrophoresis (PAGE) and electroblotted them on the polyvinylidene fluoride membranes (Millipore, USA). We then performed the immunoblotting experiments and evaluated the protein expression through Image J.

### Invasion and migration assays

We conducted the transwell invasion assay and migration assay for melanoma cells, which were previously transfected with specific molecules. After 36 h, we let the cells starve in serum-free medium for another 12 h. Next, we trypsinized them and re-seeded the cells on the top chambers of the 24-well transwell culture inserts (Corning, USA). Then, one day later, we fixed the cells in 4% paraformaldehyde for 10 min at 25 °C. For invasion assays, we coated the transwell chambers with Matrigel (BD Biosciences, NJ). We removed the non-invasive cells on the upper, and stained invasive cells on the lower, with crystal violet. In the end, we selected 5 random areas, and quantified the invaded cells by the “Multi-point” tool in Image. For migration assays, similar procedures were performed as the invasion assay, excluding the coating of Matrigel on the chamber.

### In vivo tumor xenograft assay

In the 5-week-old athymic nude mice, we inoculated lentiviral transduced A375 cells into subcutaneous spaces under the mice’s dorsal skin. Every week, we evaluated the xenografts through the measuring of their subcutaneous lengths (Ls) and widths (Ws). We calculated the in vivo tumor volumes (Vs) by *V *= l*W*W/2. The mice were then sacrificed after 5 weeks, and the xenografts would be exposed at that time.

### Statistical analysis

We presented the data as mean ± SD from three independent experimental results and processed by SPSS 17.0 (SPSS, USA). We used the Student’s paired test and one-way ANOVA to compare the variations in different groups, p < 0.05 was considered to be significant difference.

## Results

### NEAT1 is overexpressed in human melanoma tissues

**Fig. 1:**
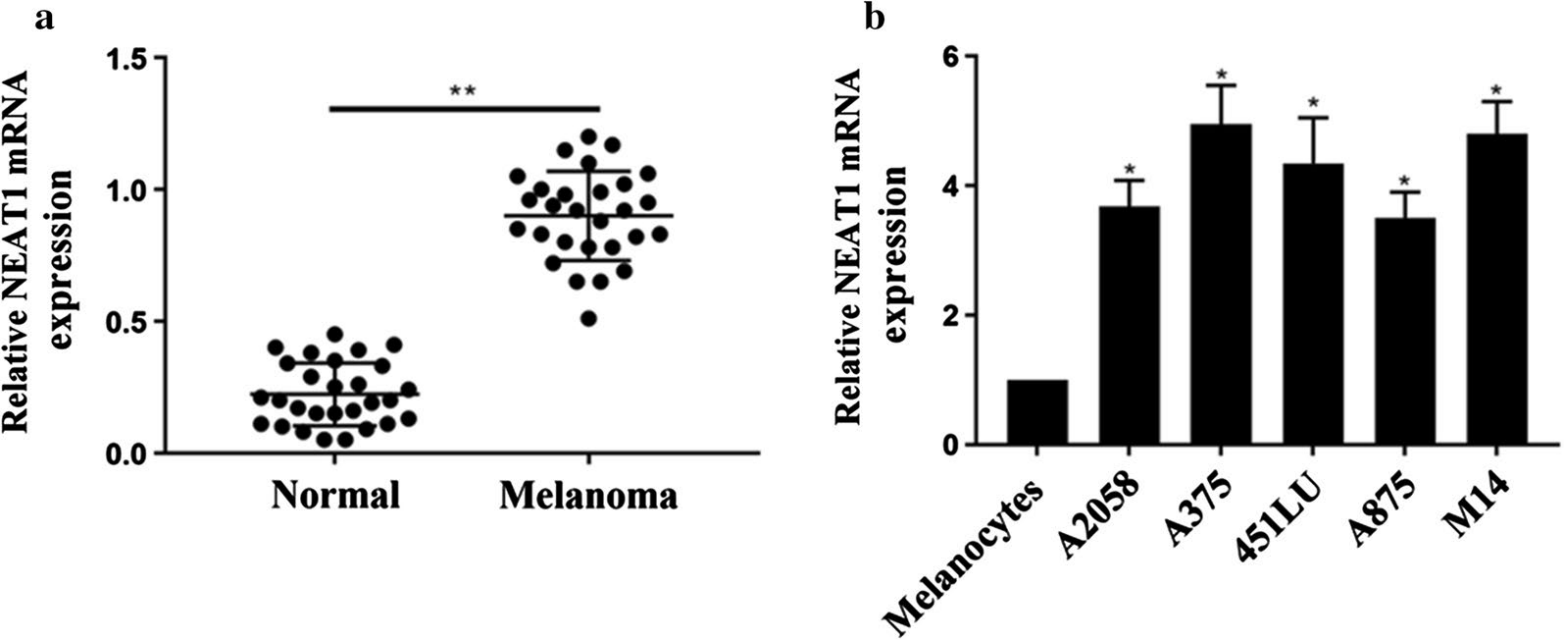
The overexpression of NEAT1 in melanoma cancer tissues. **a** NEAT1 expression level in 28 groups melanoma cancer tissues and normal tissue. **b** The expression of NEAT1 in melanocytes, and 5 melanoma cancer cell lines. *p < 0.05; **p < 0.05

We evaluated the expressions of NEAT1 in 28 groups of melanoma cancer tissues and their adjacent normal tissues by RT-PCR. Figure 1a shows the relative NEAT1 mRNA expression. We found that, compared with that in normal tissues, NEAT1 was overexpressed in melanoma cancer tissues (p < 0.05). Figure 1b shows that the expression level of NEAT1 was significantly increased in all the metastatic melanoma cell lines compared with normal human melanocytes.

### Down-regulation of NEAT1 decreased proliferation, Migration and Invasion of melanoma cancer cells

**Fig. 2:**
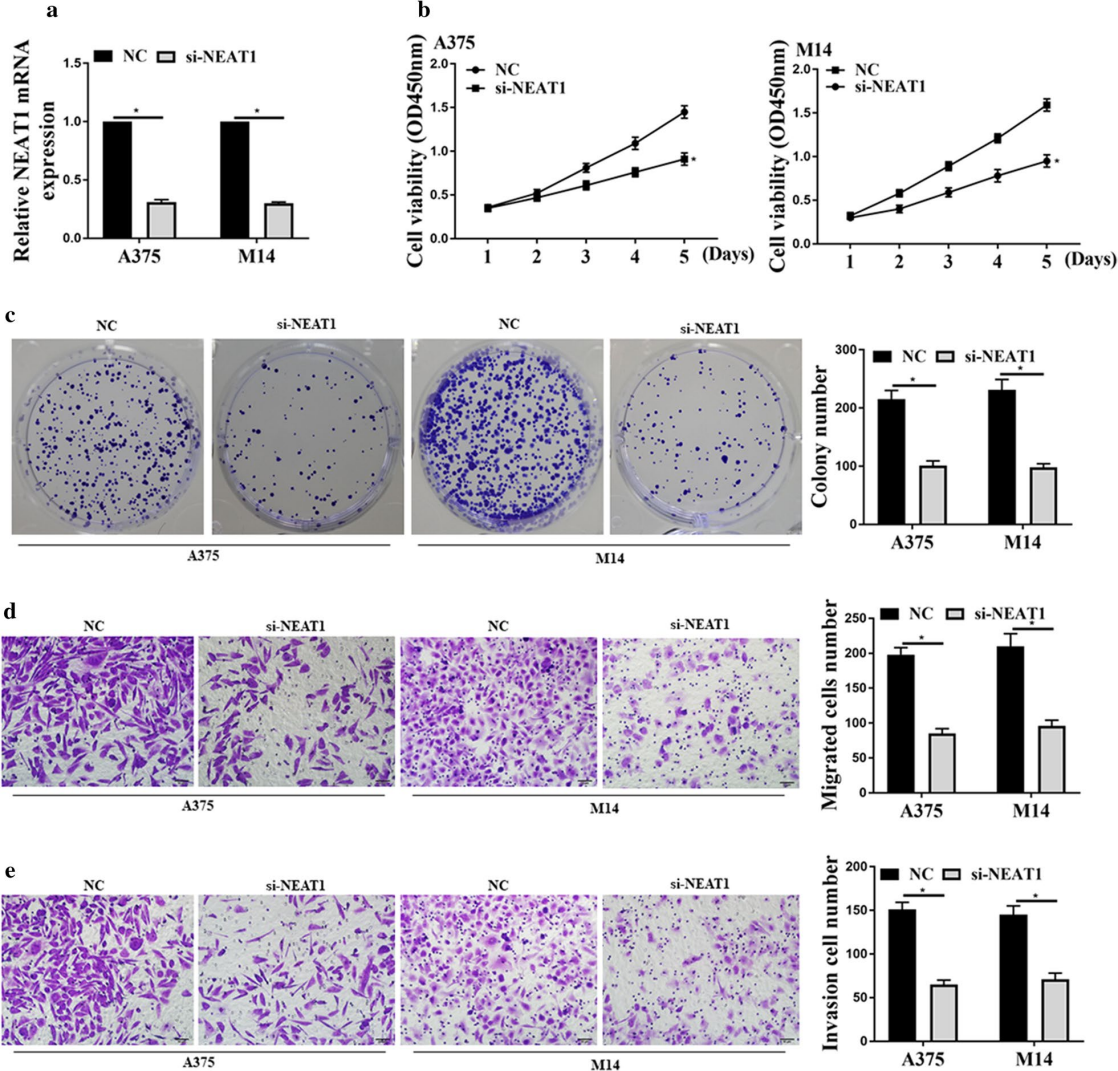
Down-regulation of NEAT1 decreased the proliferation, migration, and invasion of melanoma tumor cells. **a** The expression of NEAT1 in A375 and M14 cell lines, and **b** CCK8 analysis; **c** colony formation assays and data analysis; **d** transwell assay for cell migration and data analysis; **e** transwell assay for cell invasion and data analysis; *p < 0.05, n = 3

Firstly, we transfected the small interfering RNA of NEAT (si-NEAT1) into M14 and A375 cells. Figure 2a shows that the si-NEAT1 could down-regulate the expression level of NEAT1. Then, we performed the CCK-8 assay and colony formation assay. Figure 2b, c show that si-NEAT1 reduced the cells’ viability and colony numbers. Figure 2d, e show that the cell migration ability and invasion ability were decreased after NEAT1 downregulation. The above results confirmed that si-NEAT1 could suppress melanoma cell migration and invasion. In other words, NEAT1 was found to promote melanoma tumors’ proliferation, migration, and invasion.

### NEAT1 inhibited miR-23a-3p expression through 3′ -UTR

**Fig. 3:**
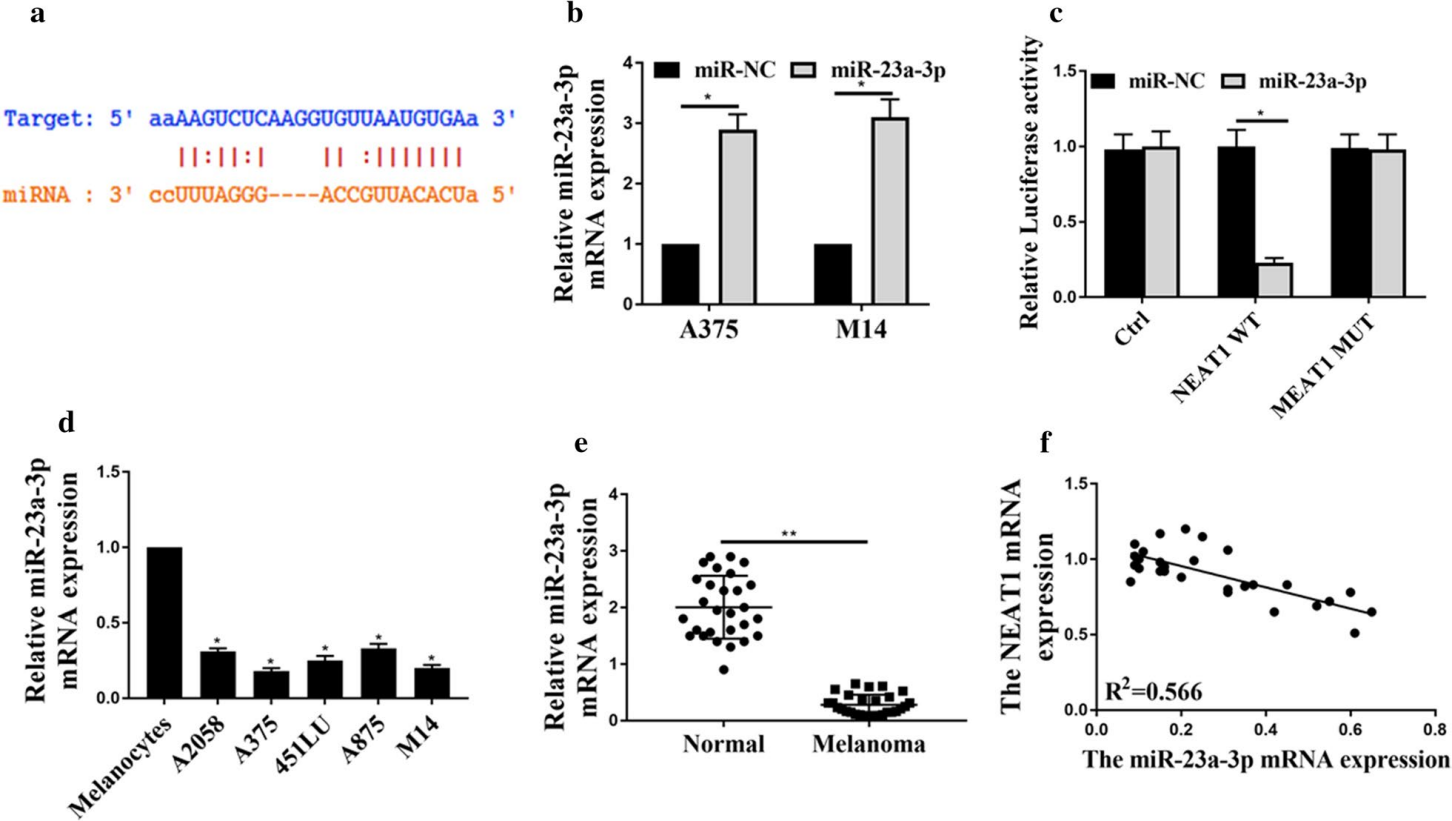
NEAT1 inhibited miR-23a-3p expression through 3′-UTR. **a** LncRNA NEAT1 was predicted to sponge miR-23a-3p by Statbase; **b** The expression of miR-23a-3p in A375 and M14; **c** The dual luciferase assay reported the expression of miR-23a-3p. **d** MiR-23a-3p expression in melanoma cancer cell lines and normal cell line. **e** MiR-23a-3p expression level in 28 groups of melanoma tissues and normal tissues; **f** Pearson’s correlation analysis for relation between the expression of NEAT1 and expression of miR-23a-3p. *p < 0.05, **p < 0.05. Ctrl: pRL-CMV vector

Bioinformatics analysis based on the online database of starBase was employed for the prediction of NEAT1′s potential target in miRNAs, miR-23a-3p. Figure 3a shows the shared binding sites at the 3′-UTR. Figure 3b shows the relative miR-23a-3p’s expression in A375 and M14. We noticed that the expression of miR-23a-3p was remarkably increased when compared with the control group. Then, we performed a dual-luciferase reporter assay to verify their paired binding. As shown in Fig. 3c, the relative luciferase activity for NEAT1 WT was dramatically decreased under the influence of miR-23a-3p, while NEA1 MUT and the control group remained the same level. Figure 3d shows that miR-23a-3p was down-regulated in all the melanoma tissue samples when we compared the results with normal cells. In addition, Fig. 3e further compared the tissues, and the phenomenon was the same that miR-23a-3p expression was down-regulated in melanoma tumors. Figure 3f shows Pearson’s correlation. It illustrates that NEAT1 was correlated negatively to miR-23 expression in human melanoma samples. The above data strongly suggest that NEAT1 could inhibit the expression of miR-23a-3p in melanoma tissue through the targeting of 3′-UTR.

### NEAT1 regulated melanoma proliferation via sponging of miR-23a-3p

**Fig. 4:**
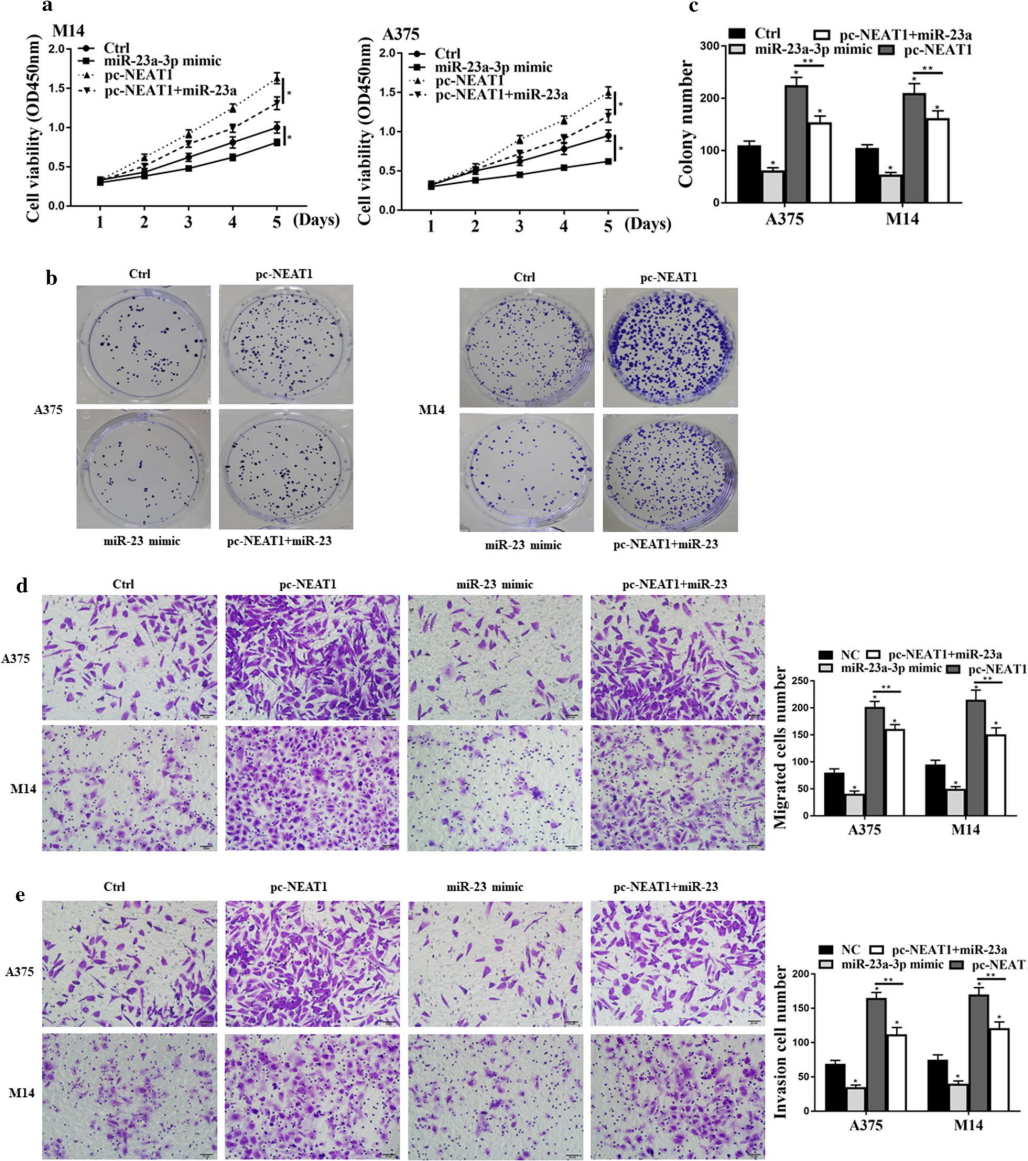
NEAT1 regulated melanoma proliferation via sponging of miR-23a-3p. **a** CCK8 assay for the cell viability; **b** cell viabilities under the microscope; **c** colony formation assays; **d** transwell assay for the investigation of cell migration; **e** transwell assay for the analysis of cell invasion; *p < 0.05, n = 3

Next, we wanted to monitor the regulation of melanoma tumors through the targeting between NEAT1 and miR-23a-3p. Figure 4a shows that the addition of miR-23a-3p always decreased cell viabilities from 0 to 5 days. Besides, the group with NEAT1 exhibited higher cell viabilities, observed from OD at 450 nm. Figure 4b, c show that pc-NEAT1 promoted the tumorigenesis of melanoma, but miR-23a mimic reversely affected lifting effects from NEAT1. Figure 4d, e show that the overexpression of NEAT1 would greatly increase cell migration and invasion. But this increasing effect would be attenuated by the transfection of miR-23a-3p mimic. As a result, the above analysis strongly suggests that NEAT1 was an oncogene in the progression of melanoma cancer through sponging with miR-23a-3p.

### MiR-23a directly targeted KLF3 and regulated KLF3 expression

**Fig. 5:**
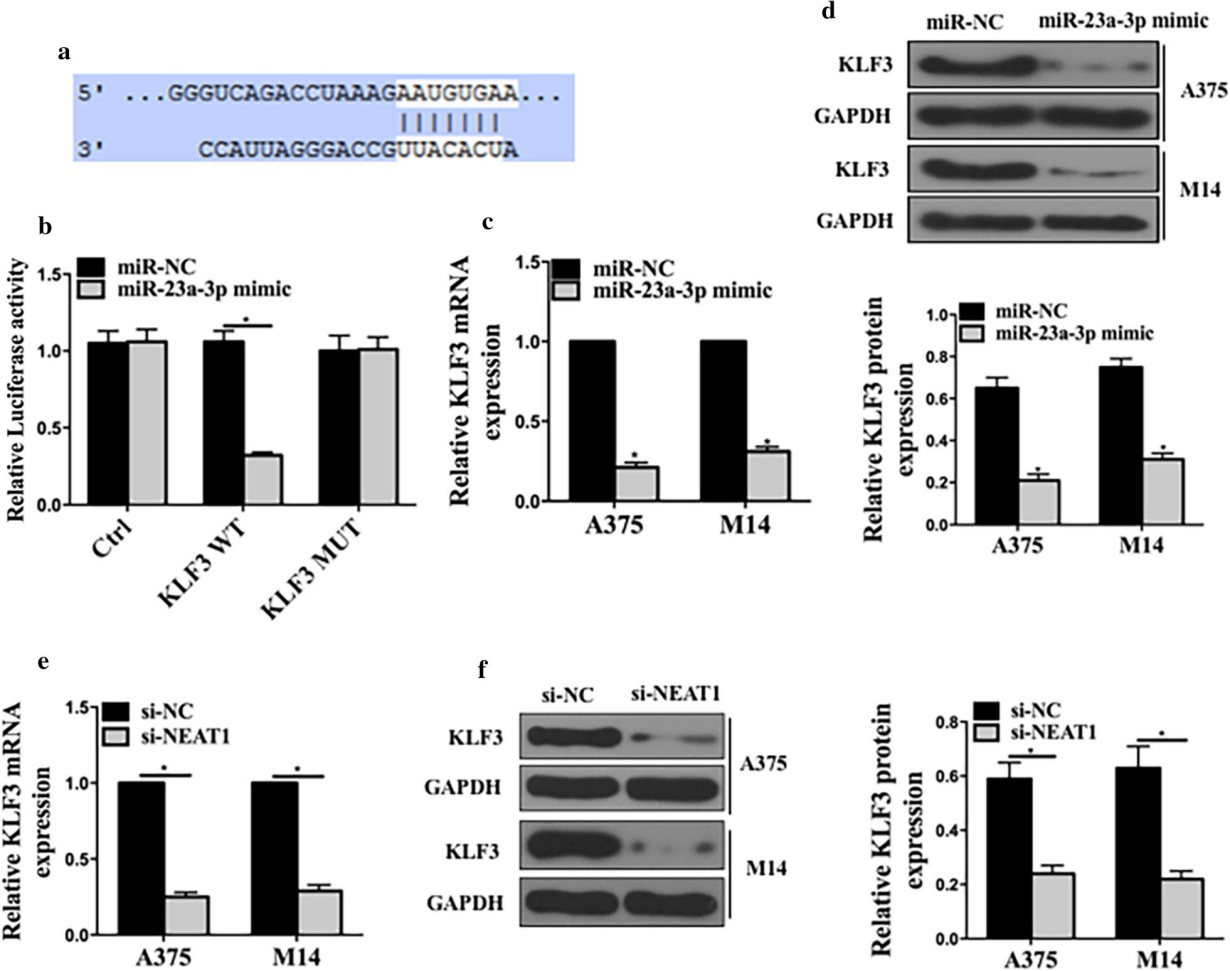
MiR-23a directly targeted KLF3 and regulated its expressions. **a** The sequence prediction on the targeting between miR-23a-3p and KLF3. **b** The dual luciferase assay for the examination of the miR-23 expression. **c** The mRNA expression of KLF3 mRNA in A375 and M14; **d** the protein expression of KLF3 in A375 and M14; **e** KLF3 mRNA expressions in cells transfected with si-NC or si-NEAT1; **f** Protein expressions in cells transfected with si-NC and si-NEAT1. *p < 0.05, n = 3. Ctrl: pRL-CMV vector

We performed bioinformatics analysis (Targetscan) to predict the potential target genes, and KLF3 was selected. Figure 5a shows the predicted shared binding sites at the 3′-UTR. Then, we performed a dual-luciferase reporter assay to verify this paired binding (Fig. 5b). Figure 5c, d show that miR-23a-3p mimic could decrease the expression of KLF3 in mRNA levels and protein levels. These experiments further confirmed that miR-23a-4p directly targeted KLF3 and down-regulated its expressions.

### NEAT1/miR-23a axis regulated melanoma proliferation, migration and invasion via KLF3

**Fig. 6:**
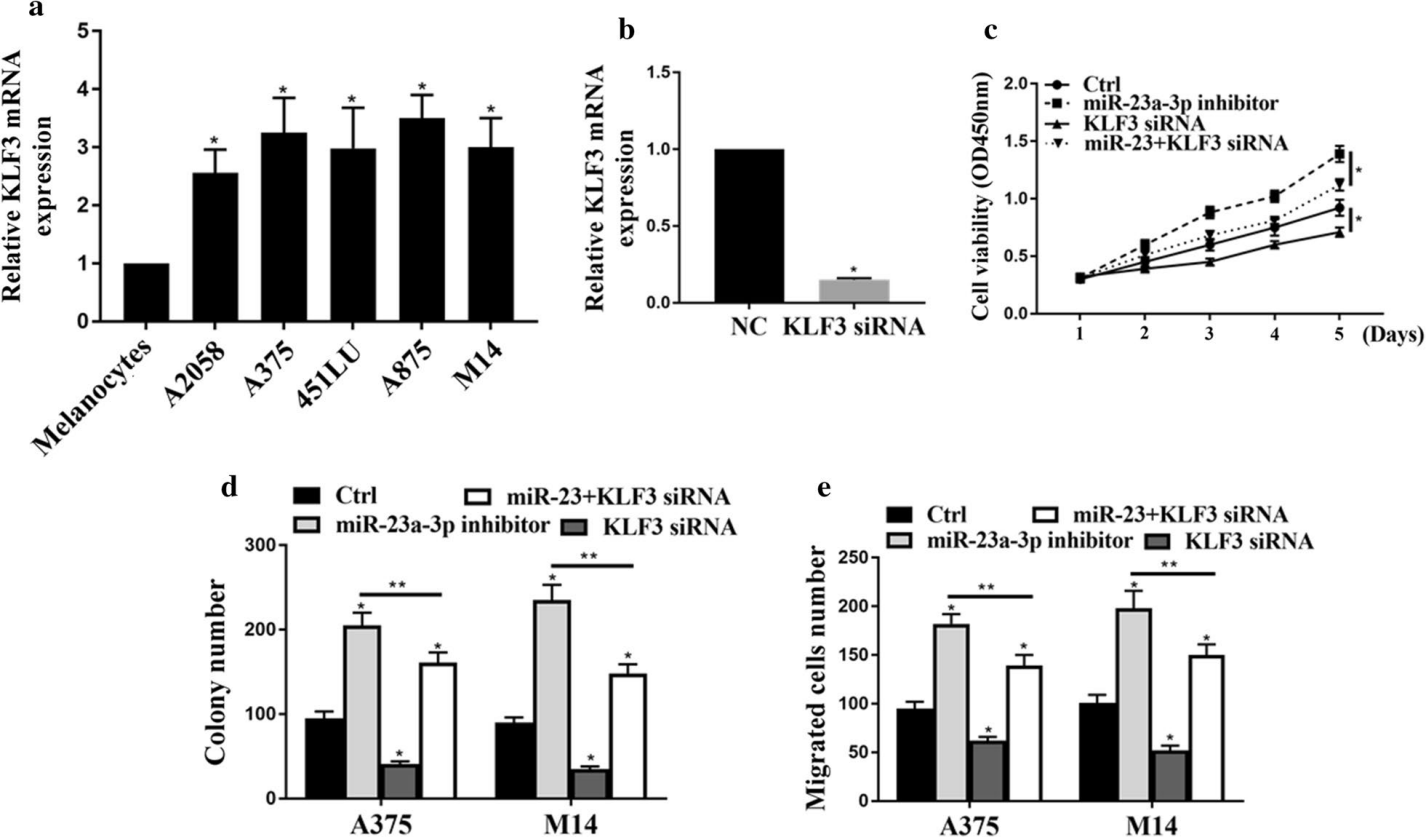
NEAT1/miR-23a axis regulated cell proliferation, migration and invasion via KLF3 in melanoma cell lines. **a** Relative mRNA expression of KLF3 in melanoma cell lines. **b** The expression of KLF3 for KLF3 si-mRNA in A375 and M14 cells. **c** CCK8 assays for cell viability; **d** colony formation analysis; **e** transwell analysis of cell migration; *p < 0.05, **p < 0.05. n = 3

Firstly, we measured the expression of KLF3 in melanoma cell lines. Figure 6a shows that KLF3 was up-regulated in melanoma cell lines, compared with normal human melanocytes. Figure 6b investigated the KLF3′s mRNA expression melanoma mediated by KLF3 si-RNA. We found that KLF3 si-RNA significantly reduced the expressions of KLF3 in both A374 and M14. Figure 6c illustrated that miR-23a-3p inhibitor could promote cell viabilities, but KLF3 siRNA inhibited the cell viabilities. Figure 6d shows the colony formation results. We found that miR-23a-3p induced cell proliferation, but these effects were latterly attenuated by KLF3 siRNA. Figure 6e, f indicated that miR-23a-3p could increase cell migration and invasion, but these effects would be attenuated by KLF3 siRNA.

### Decreased NEAT1 inhibited the growth of xenograft tumor in vivo

**Fig. 7:**
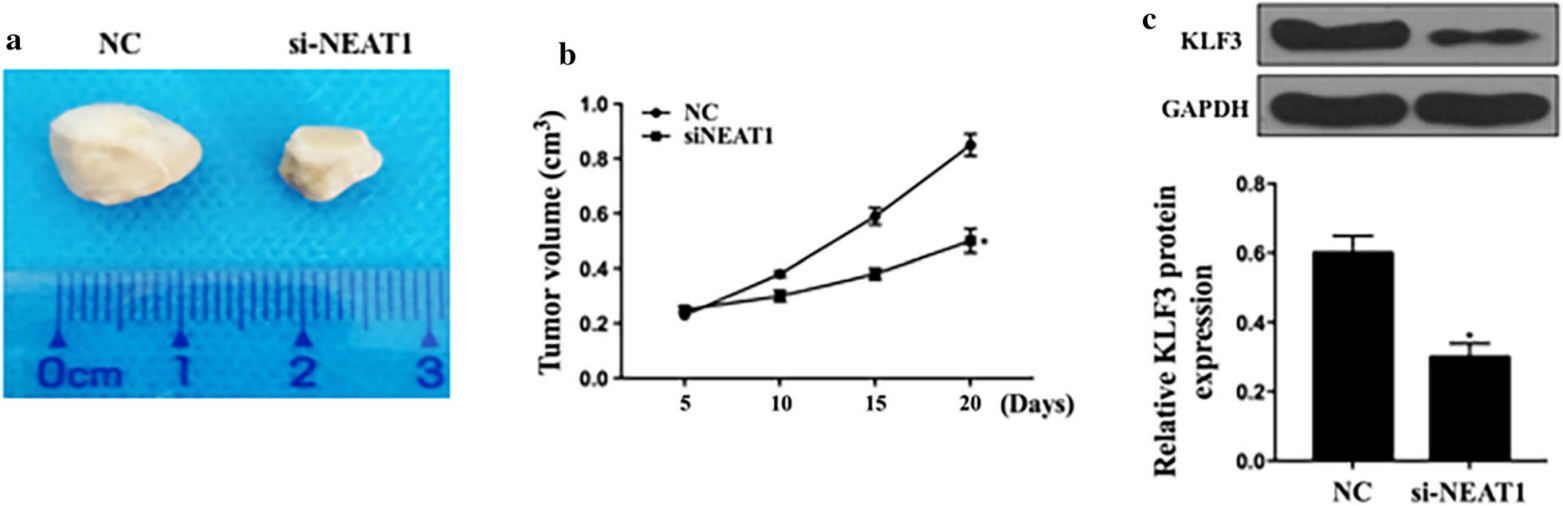
Knockdown of NEAT1 inhibited the growth of xenograft tumor in nude mice. **a** Pictures of tumors grown in the silencing of NEAT1; **b** tumor volume growth curve from 0 to 20 days; **c** the relative KLF3 mRNA expression levels in the silencing of siRNA; *p < 0.05

In vivo experiments were performed to investigate the effect of NEAT1 on tumor growth. We transfected the cells with NC or si-NEAT1 and injected them into the BALB/c nude mice. Figure 7a shows that the si-NEAT1 remarkably decreased the tumor volumes. Figure 7b shows the tumor growth curve in different days, with si-NEAT1 tumors smaller than that from the control group. We also measured KLF3 expression in KLF3 si-mRNA, and we found that the silencing of KLF3 significantly lowered the expression. The above results further conformed that si-NEAT1 inhibited the growth of xenograft tumor in vivo.

## Discussions

There have studies reported that NEAT1 was overexpressed during cancer development, including papillary thyroid cancer [17] and non-small cell lung cancer [16]. In 2019, Wu recently found that NEAT1 was involved in the axis of NEAT1/has-miR-98-5p/MAK6, and its over-expression in tumor cells promoted lung cancer development [16]. Besides, H. Tan found that NEAT1 could modulate miR-506, and thus involved in the gastric cancer development [8]. Although there are not many studies support its role in melanoma cancer, our study provided evidence about its expression effect in this disease. The RT-PCR analysis revealed that the expression of NEAT1 was remarkably increased in all metastatic melanoma cell lines when compared with normal cells. In addition, the CCK-8 assay and colony formation assay confirmed that si-NEAT1 greatly reduced the viability of cells, as well as the capabilities in invasion and migration. Our findings are thus in consistent with the previous reports that the expression of NEAT1 is positively related with cancer cell proliferation.

Accumulated studies have suggested that lncRNAs could sponge some specific types of miRNA and thus regulate tumorigenesis [5–7, 11]. To our best knowledge, no one has investigated whether NEAT1 could target miR-23a-3p. We made a hypothesis that these two non-coding RNAs could sponge each other. Through gene database prediction, we found that miR-23a-3p is a target of NEAT1. Besides, the CCK-8 assay, colony formation assay, and transwell assay further proved that the expression of miR-23a-30 was inhibited by the adding of NEAT1.

It has been stated that miR-23a-3p could act as a suppressor in the proliferation, migration, and invasion of tumor cells [20]. Researchers have revealed that miR-23a-3p could lower the speed and rate of oral squamous cell proliferation [20]. It suppressed the growth of OSCC tumor through the targeting of FGF2. There are inadequate studies about its role in melanoma cancer development, but many other microRNAs, such as miR-579-3p [29], miR-204-5p [30], and miR-16 [4], have been discovered to regulate the capabilities of proliferation, migration, and invasion in melanoma cells. Our study revealed that miR-23a-3p, as well as the previously reported microRNAs, could suppress the melanoma cell growth. In the CCK-8 assays, we found that the addition of miR-23a-3p mimic could greatly reduce the cell viabilities and colony numbers of melanoma cell lines. However, this effect was later attenuated by the interaction with lncRNA-NEAT1.

To investigate the attenuation effect between NEAT1 and miR-23a-5p, we further conducted a hypostasis that NEAT1 could sponge miR-23a-5p and lower the effect that was caused by miR-23a-5p. Previous studies have found the sponging between lncRNAs and microRNAs could regulate cancer cell proliferation. For example, Sun et al. had discussed that the lncRNA MALAT1 could sponge miR-183 and targeted ITGB1 [11]. This phenomenon promoted the development of melanoma cancer. We are the first to unveil the masks that lncRNA NEAT1 could sponge miR-23a-3p, and this regulation could result in the proliferation of melanoma cancer. Our data and analysis in the migration/invasion assay were in consistency with previous findings.

The KLF3 siRNA gene could also repress the expression of some specific genes. For example, in 2018, KLF3 was demonstrated to participate with miR326 to form a regulatory axis and suppress the progression of lung cancer cells [26]. We examined the expressions of KLF3 in melanoma cell lines and found that miR-23a-3p could directly target and decrease the expression of KLF3. Besides, the transwell assay indicated that the increasing of cell proliferation by miR-23a-3p could be attenuated by KLF3 siRNA. The interaction between KLF3 and miR-23a-3p relies on their targeting effect. Through regulation in the expression of KLF3 by miR-23a-3p, NEAT1/miR-23a mediated melanoma proliferation, migration, and invasion.

## Conclusions

The above data, comparison, and analysis supported our hypostasis that oncogene lncRNA-NEAT1 could sponge miR-23a-3p and functions via the expression of KLF3. This axis could together regulate melanoma cancer proliferation. We believe this finding could provide a new therapeutic strategy for melanoma skin cancer.

## Data Availability

The analyzed data sets generated during the study are available from the corresponding author on reasonable request.
